# Investigating the longitudinal bi-directional relationship between self-reported restrictive eating behaviours and sleep in UK adolescents within the Millennium Cohort Study

**DOI:** 10.1007/s00787-025-02641-9

**Published:** 2025-01-28

**Authors:** Marie-Christine Opitz, Giulia Gaggioni, Nora Trompeter, Francisco Diego Rabelo-da-Ponte, Sylvane Desrivières, Nadia Micali, Ulrike Schmidt, Helen Sharpe

**Affiliations:** 1 University of Edinburgh, Edinburgh, United Kingdom; 2 University College London, London, United Kingdom; 3 King’s College London, London, United Kingdom; 4 Ballerup Psychiatric Centre Eating, Copenhagen, Denmark; 5 South London and Maudsley NHS Foundation Trust, London, United Kingdom

**Keywords:** Sleep, Disordered eating, Millennium cohort study, Longitudinal research

## Abstract

**Objective:**

This study aimed to investigate the longitudinal bi-directional relationship between self-reported restrictive eating behaviours and sleep characteristics within a sample of UK adolescents from the Millennium Cohort Study (MCS).

**Method:**

Using a Structural Equation Modelling approach, the present study investigated the prospective associations between individual sleep behaviours (e.g., sleep timing, sleep onset latency, social jetlag) at age 14 and restrictive eating behaviours at age 17. Moreover, the association between restrictive eating behaviours (age 14) and self-reported sleep quality (age 17) was tested. A mediation analysis was conducted to explore the role of depressive symptoms in these relationships. In total, *N* = 6,041 young people provided self-report data at both timepoints (sweep 6 & 7) and a subsample of *N* = 2,164 additionally provided diary data on their sleep behaviours over two separate 24 h periods.

**Results:**

Sleep indicators at age 14 did not significantly predict changes in restrictive eating behaviours across time. However, engagement in restrictive eating behaviours at age 14 significantly predicted poorer self-perceived sleep quality three years later (β = 0.06, SE = 0.01, *p* <.01). Depressive symptoms fully mediated this relationship (indirect effect: β = 0.05, SE = 0.04, *p* <.001).

**Discussion:**

The present study provides evidence for a prospective positive association between restrictive eating behaviours and subsequent poorer sleep quality in a large, general population sample. Findings of the mediation analysis suggest mood as a potential target for tertiary prevention when addressing restrictive eating behaviours as an eating disorder risk factor in adolescents.

**Supplementary Information:**

The online version contains supplementary material available at 10.1007/s00787-025-02641-9.

## Introduction

Within the last decades, shortening sleep duration and increasing sleep problems have been observed globally (e.g., for changes in sleep across the 2010s, see [1]), with adolescents being uniquely affected by sleep difficulties due to biological (e.g., delay in sleep onset) and social (e.g., lack of sleep hygiene) factors [2]. The latest international Health Behaviours in School-aged Children Survey [3] showed that one in four 11- to 15-year-olds report difficulties in getting to sleep, with English 15-year-olds exhibiting the highest amount of sleep difficulties across all 44 countries. The amount and increase in sleep difficulties are concerning, considering sleep’s crucial role in adolescent functioning [4] and association with various health outcomes [2].

Meanwhile, increasing rates of weight-control behaviours have been identified in UK adolescent populations, potentially due to a heightened awareness of anti-obesity messaging [5]. While increasingly more common, this development is equally concerning, as adolescent girls’ dieting behaviours have been found to be predictive of later binge eating, extreme weight control behaviours, and reported eating disorders (EDs) [6], with heightened ED risk not only being associated with individuals’ past-year dieting experiences, but also friends’ dieting behaviours [7]. Similarly, even though found to be less common, adolescents’ intentions to lose weight have been associated with a higher likelihood of being classified as at risk for an ED [8] and disordered eating [9].

As reflected in the high prevalence rates of sleep problems and dieting behaviours, adolescents have a heightened vulnerability for the onset of both eating and sleep difficulties [10]. Thereby, sleep and eating habits have been shown to be related [11], with clinical research particularly focusing on obesity-related factors, such as the relationship between sleep and weight loss maintenance [12, 13]. Yet, considering that disordered eating has been linked to sleep difficulties through a variety of mechanisms, such as overstimulation (excessive exercise), increased hunger and fatigue (dietary restriction), or excessive napping (meal avoidance) [14], there is a need for more research investigating the relationship between sleep and restrictive eating behaviours (such as dieting).

Since a bi-directional relationship between eating patterns and sleep is possible, if not likely [15], there appears to be a clear need for longitudinal investigations. Large-scale longitudinal research approaches to examine the relationship between sleep and restrictive eating behaviours might also shed further light on the role of potential mediating factors of this relationship. A better understanding of *how* sleep and restrictive eating behaviours are linked could inform guidelines for clinical practice to identify where and when to intervene [16], before clinical levels of behaviours are reached. For instance, depressive symptoms could be the result *of* and precursor *for* both restrictive eating behaviours and altered sleep [5, 14].

Taking into account the concerning high prevalence of sleep difficulties in UK adolescents, as well as increasing rates of weight-change behaviours in both UK girls and boys, the present study addresses current research gaps regarding potential associations between sleep and restrictive eating behaviours in adolescents. A better understanding of the relationship between sleep and restrictive eating behaviours might help target preventative interventions for those at risk of developing an ED as well as prevention of symptom relapse in ED recovery [14]. Furthermore, identifying potential risk factors for poorer sleep will aid in addressing sleep-related emotional and behavioural problems, as well as improving overall health [17] and tertiary ED prevention approaches.

### Research questions

1. Do sleep indicators (measured at age 14) predict higher levels of restrictive eating behaviours (measured at age 17), while controlling for baseline levels of restrictive eating?
2. Do restrictive eating behaviours (measured at age 14) predict perceived lower sleep quality (measured at age 17), while controlling for baseline levels of sleep indicators?
3. Do depressive symptoms (measured at age 14) mediate the relationship between restrictive eating behaviours and sleep?

Based on previous research findings, bi-directional associations between sleep characteristics and restrictive eating behaviours were expected, with depressive symptoms mediating this relationship.

## Methodology

This project is a secondary data analysis of the Millennium Cohort Study [18]. Since 2001, eight data sweeps have been collected. At the first data collection point, 18,818 cohort children and 18,552 families were included. Further information about the MCS study characteristics and design can be found in Plewis and colleagues [19]. A study protocol for the current project was pre-registered on the Open Science Framework (OSF, 10.17605/OSF.IO/S92MT*)* prior to data analysis and all analysis codes uploaded on GitHub (https://github.com/M-COpitz/MCS_Sleep_Disordered_Eating.git*).* This study received ethical approval from the University of Edinburgh Research Ethics Committee (Reference: 22-23CLPS066). Further information on available data and measures is provided in the supplementary material.

### Participants

MCS data from sweep 6 (*N* = 15,414 children aged 14, assessed 2015) and sweep 7 (*N* = 14,438 children aged 17, assessed 2018) were used for data analysis and accessed through the UK Data Service. All cohort children who self-reported on restrictive eating behaviours and sleep outcomes at both time points were included in the analyses. For twin pairs, one twin was randomly selected for inclusion. This led to a final analytic sample of *N* = 6,041. At age 14, a self-selected subsample of participants additionally reported on their time use on two randomly assigned assessment points. Those reporting on their time-use without missing data on both days were included for all sub-sample analyses (*N* = 2,164).

### Measures

#### Restrictive eating behaviours

Restrictive eating behaviours were assessed using three available indicators: dietary restriction (*“Have you ever eaten less food, fewer calories, or foods low in fat to lose weight or to avoid gaining weight?”*, 1 = yes, 0 = no), exercise for weight control (*“Have you ever exercised to lose weight or to avoid gaining weight?”*, 1 = yes, 0 = no), and intention to lose weight (“Which of the following are you trying to do about your weight?”, 1 = lose weight, 0 = gain weight, stay the same weight, I am not trying to do anything about my weight). These items were assessed at age 14 and 17, with the former two items referring to life-time behaviours up until the age of 14 at the first assessment point, and recent experiences (previous 12-months) at age 17.

#### Sleep

At age 14, the MCS assessed adolescent-reported bed-/wake time categories, and single-items on sleep onset timings and difficulties with awakening during the night. To depict *problems* with sleep and to replicate previous approaches, the following sleep variables were created: participants’ typical sleep durations on school days and school-free days were categorised as ≤ 8 h, 8–9 h, 9–10 h, and ≥ 10 h, to align categories with recommendations of the American Academy of Sleep Medicine for adolescent populations (13–18 years) [17]. The median wake-up and bedtimes were used to divide participants in two groups. As sleep variables were already pre-categorised and this study aimed to assess associations with problematic sleep behaviours, dichotomous variables were created to depict earlier/later (i.e., potentially problematic) wake-up (< 7.30am school days, < 10.30am school-free days) and bedtime categories (> 10.30pm school days, > 11.30pm school-free days). Social jetlag, the discrepancy between individuals’ biological and social clocks [20], was calculated as:

Social Jetlag = midpoint of sleep on school-free days (biological time) – midpoint of sleep on school days (social time).

In line with clinical recommendations [21] and previous approaches [22], sleep onset latency was defined as presence (> 30 min) or absence (≤ 30 min) of sleep onset difficulties. Wake After Sleep Onset (WASO) was equally dichotomised (presence = difficulties “all of the time”, “most of the time”, or “a good bit of the time”) [22]. Sleep problems were thereby contrasted with a lack of sleep problems in accordance with previous studies [22], to investigate links between restrictive eating and problematic sleep behaviours.

Time use data (TUD) captured a subsample’s activities during two 24 h periods (during the week and during the weekend), recorded within 10 days of the interview visit. Three variables were created for both weekdays and weekend days: 24-hour sleep duration (all sleep reported during each 24 h period), awakening during the night (the presence or absence of reporting at least one incidence of awakening between 8pm and 5.30am to avoid capturing naps and typical wake-up times), and day-time naps (presence or absence of naps during the day, with a nap being defined as any sleeping period between 9am and 7pm, which occurred after being awake for at least 60 min).

Participants’ self-rated overall sleep quality (*“During the past month, how would you rate your sleep quality overall? Would you say it has been…”*, “Very good”, “Fairly good”, “Fairly bad”, “Very bad”) was assessed at age 17.

#### Symptoms of depression

Symptoms of Depression were assessed via the Short Moods and Feelings Questionnaire (SMFQ), a 13-item measure with sum-score values ranging from 0 to 23 [23]. Adolescents (age 14) self-reported how they were feeling/acting during the previous two weeks (e.g., “I felt miserable or unhappy”; “not true”, “sometimes”, “true”). Good internal reliability of the SMFQ was previously found across time within a general adolescent population [24]. Within the present study, Cronbach’s alpha was 0.93 (ω = 0.93). For all relevant analyses, depressive symptoms were modelled as one latent factor.

#### Covariates

Relevant sociodemographic covariates were included as provided in the MCS. These were participants’ sex (female or male), ethnicity (White, mixed, Indian, Pakistani or Bangladeshi, Black or Black British, other ethnic group), weight category (adjusted BMI categories formulated by the UK90 [25]), and household income (equivalised income quintiles by country). Seasonality was accounted for in all TUD analyses (spring, summer, autumn, and winter).

### Analyses

All analyses were conducted using R version 4.3.1 (see supplementary material for packages used). Patterns of item missingness for predictors and outcomes were explored using Little’s MAR test and the ‘naniar’ package. At data sweep 7, 55.2% of the full initial MCS sample (*N* = 19,243) were considered ‘productive’ survey responders. For more information on participant response rates, see Ploubidis and Mostafa [26] for sweep 6 and Ipsos MORI [27] for sweep 7. Missing data and survey weighting approaches for this project are outlined in the supplementary material.

Associations between relevant variables were calculated using chi-square tests, phi coefficients (dichotomous variables), Cramer’s V (categorical-dichotomous associations), and point-biserial correlation coefficients (continuous-categorical associations). To assess the latent factor structure as well as the extent to which the specified latent construct measured restrictive eating behaviours consistently across time, the model’s longitudinal measurement invariance was evaluated following the approach outlined in Mackinnon et al. [28] (findings presented in supplementary material).

To address the outlined research questions, a series of regression analyses were conducted within a structural equation modelling (SEM) framework. Restrictive eating behaviours were operationalized as a latent factor, and all sleep variables were added as manifest variables. First, sleep indicators (measured at age 14) were individually modelled as predictors of restrictive eating behaviours (measured at age 17), controlling for restrictive eating behaviours at age 14. Second, restrictive eating behaviours at age 14 was specified as a predictor for perceived sleep quality (measured at age 17), controlling for all sleep indicators at age 14. All analyses that included TUD were conducted on the available sub-sample. Due to the non-continuous nature of the indicator variables, the robust weighted least squares (WLSMV) estimator was specified to estimate robust standard errors. SEM mediation models were tested for all significant longitudinal models, specifying the mediator as measured at baseline (age 14). A value of *p* <.05 was used to determine significance. To account for multiple testing, the Benjamini-Hochberg correction with a false discovery rate of 0.05 was used to adjust p-values in relevant analyses.

## Results

Demographic information for both samples are shown in Table 1.

**Table 1:** Overview of participant characteristics

Participant Characteristics	Analytic Sample (*N* = 6,041)		Time-Use Data Subsample (*N* = 2,164)
Self-Reported Sex, n (%)			
Female	3,404 (56.35%)		920 (42.51%)
Male	2,637 (43.65%)		1,244 (57.49%)
Self-Reported Ethnicity, n (%)			
White	4,926 (81.56%)		1,876 (86.69%)
Pakistani and Bangladeshi	362 (5.99%)		76 (3.51%)
Mixed	266 (4.40%)		80 (3.7%)
Black/Black British	176 (2.91%)		36 (1.66%)
Indian	171 (2.83%)		60 (2.77%)
Others (incl. Chinese)	141 (2.32%)		36 (1.66%)
Parent-Reported Household Income, n (%)			
“lower quintile”	735 (12.17%)		154 (7.12%)
“second quintile”	846 (14%)		230 (10.63%)
“third quintile”	1,148 (19%)		387 (17.88%)
“fourth quintile”	1,556 (25.76%)		630 (29.11%)
“highest quintile”	1,756 (29.07%)		763 (35.26%)
UK90 BMI Categories, n (%)			
“underweight”	94 (1.57%)		38 (1.77%)
“healthy weight”	3,783 (63.31%)		1,422 (66.14%)
“overweight”	929 (15.55%)		332 (15.44%)
“obese”	1,169 (19.56%)		358 (16.65%)
Missing	66 (0.01%)		14 (< 0.01%)
Age 14 Sleep Duration (School Day), n (%)			Age 14 TUD Sleep Duration (Week), n (%)
≤ 8 h	2,538 (42.01%)		417 (19.27%)
> 8–9 h	2,382 (39.43%)		648 (29.94%)
> 9–10 h	1,019 (16.87%)		558 (25.79%)
> 10 h	102 (1.69%)		541 (25.00%)
Age 14 Sleep Duration (School-Free Day), n (%)			Age 14 TUD Sleep Duration (Weekend), n (%)
≤ 8 h	285 (4.72%)		241 (11.14%)
> 8–9 h	834 (13.81%)		282 (13.03%)
> 9–10 h	1,820 (30.13%)		443 (20.47%)
> 10 h	3,102 (51.35%)		1,198 (55.36%)
Age 14 Sleep Onset Latency, n (%)			Age 14 Daytime Nap (Week), n (%)
Yes	2,054 (34%)		108 (4.99%)
No	3,987 (66%)		2,056 (95.01%)
Age 14 Wake After Sleep Onset, n (%)			Age 14 Daytime Nap (Weekend), n (%)
Yes	1,029 (17.03%)		102 (4.71%)
No	5,012 (82.97%)		2,062 (95.29%)
Age 14 Social Jetlag, M (SD)	1.92 (0.81)		Age 14 Nighttime Awakening (Week), n (%)
hh: mm, M (SD)	1h55min (49 min)		Yes: 89 (4.11%)
	Skew = 0.15		No: 2,075 (95.89%)
	Kurtosis = 0.05		
Age 14 Wake-Up Time (School Days), n (%)			Age 14 Nighttime Awakening (Weekend), n (%)
< 7.30am	2,718 (44.99%)		Yes: 84 (3.88%)
≥ 7.30am	3,323 (55.01%)		No: 2,080 (96.12%)
Age 14 Wake-Up Time (School-Free Days), n (%)			
< 10.30am	3,114 (51.55%)		
≥ 10.30am	2,927 (48.45%)		
Age 14 Bedtime (School Days), n (%)			
≤ 10.30pm	4,558 (75.45%)		
> 10.30pm	1,483 (24.55%)		
Age 14 Bedtime (School-Free Days), n (%)			
≤ 11.30pm	4,273 (70.73%)		
> 11.30pm	1,768 (29.27%)		
Age 17 Overall Sleep Quality, n (%)			
“Very good”	945 (15.64%)		
“Fairly good”	3,197 (52.92%)		
“Fairly bad”	1,470 (24.33%)		
“Very bad”	429 (7.10%)		

### Restrictive eating behaviours

Within the full analytic sample, 42.26% (*n* = 2,553) of 14-year-olds reported an intention to lose weight, 44.71% (*n* = 2,701) reported ever having used dietary restriction to lose weight, and 59.82% (*n* = 3,614) having used exercise to lose weight. At age 17, 48.05% (*n* = 2,903) reported an intention to lose weight, 52.28% (*n* = 3,158) dietary restriction for weight loss within the past year, and 62.75% (*n* = 3,791) the use of exercise to lose weight. Supplementary Table 4 illustrates the proportions by sex, UK90 BMI-adjusted categories, household income quintiles, and ethnicity.

### Sleep characteristics

Table 1 illustrates descriptive characteristics of all sleep variables within the full analytic and the TUD sub-sample. The different sleep duration measurement approaches produced different category sizes, especially for school day/week measurements. Around a third of 17-year-olds included in this study reported either “fairly bad” or “very bad” overall sleep quality.

### Correlations between sleep and restrictive eating behaviours variables

A full overview of correlations between sleep and restrictive eating behaviour variables is provided in Supplementary Tables 5–9. Amongst sleep indicators, WASO displayed the strongest associations with weight loss intention (*r* =.14, 95% CI [0.12, 0.16]), dietary restraint (*r* =.16, 95% CI [0.14, 0.18]), and excessive exercise (*r* =.11, 95% CI [0.09, 0.14]). TUD sleep assessments were not significantly associated with any of the restrictive eating indicators, except for daytime napping and dietary restriction at age 14 (*r* =.07, 95% CI [0.05, 0.10]). The strongest correlation between overall poorer self-perceived sleep quality (age 17) and restrictive eating behaviours was identified for weight loss intention (age 14: *r* =.10, 95% CI [0.07, 0.12]; age 17: *r* =.12, 95% CI [0.10, 0.15]) and dietary restriction (age 14: *r* =.11, 95% CI [0.09, 0.14]; age 17: *r* =.11, 95% CI [0.09, 0.14]).

### Cross-sectional associations between sleep and restrictive eating behaviours

Supplementary Tables 10–12 illustrate explorative findings from cross-sectional models investigating individual sleep characteristics as predictors for restrictive eating behaviours within each timepoint. Although effect sizes were small, all age 14 self-reported sleep characteristics (except for school-free day wake-up time) were significantly associated with restrictive eating behaviours. Higher restrictive eating behaviours were thereby associated with shorter sleep duration, presence of sleep onset latency and WASO, later sleep timing on school-free nights, wake-up times earlier than 7.30am on school days, and later bedtimes. Equally, self-perceived poorer overall sleep quality was significantly associated with higher restrictive eating behaviours at age 17 (β = 0.09, SE = 0.02, *p* <.001). In contrast, none of the TUD sleep variables exhibited any significant associations with restrictive eating behaviours.

### Longitudinal associations between sleep and restrictive eating behaviours

Findings from longitudinal analyses are illustrated in Tables 2, 3 and 4. The model fit for all models specifying sleep indicators as predictors for subsequent restrictive eating behaviours were mixed, but improved once a covariance between both BMI and sex with restrictive eating behaviours (age 14) was specified (CFI = 0.96, TLI = 0.94, RMSEA = 0.07, SRMR = 0.05). None of the sleep indicators significantly predicted restrictive eating behaviours at age 17.

**Table 2:** Overview SEM regression outcomes for self-reported sleep indicators (Age 14) predicting restrictive eating behaviours (Age 17) (*N* = 5975)

Model	Predictor	Beta	SE	*p*	*p* (adjusted)*	Fit Indices
Model 1	Sleep Duration (School Day)	− 0.01	0.02	0.3	0.68	χ^2^(35, 5975) = 1217.23, CFI = 0.96, TLI = 0.94, RMSEA = 0.07, SRMR = 0.05
	**Restrictive Eating Behaviours (age 14)**	**0.4**	**0.03**	**< 0.001**		
	**Sex**	**0.15**	**0.03**	**< 0.001**		
	Ethnicity	0.004	0.01	0.76		
	**BMI**	**0.14**	**0.02**	**< 0.001**		
	Income	− 0.01	0.01	0.49		
Model 2	Sleep Duration (School-Free Day)	− 0.01	0.02	0.49	0.82	χ^2^(35, 5975) = 1227.55, CFI = 0.96, TLI = 0.94, RMSEA = 0.07, SRMR = 0.05
	**Restrictive Eating Behaviours (age 14)**	**0.4**	**0.03**	**< 0.001**		
	**Sex**	**0.15**	**0.03**	**< 0.001**		
	Ethnicity	0.004	0.01	0.77		
	**BMI**	**0.14**	**0.02**	**< 0.001**		
	Income	− 0.01	0.01	0.46		
Model 3	Sleep Onset Latency	− 0.003	0.03	0.78	0.82	χ^2^(35, 5975) = 1217.23, CFI = 0.96, TLI = 0.94, RMSEA = 0.07, SRMR = 0.05
	**Restrictive Eating Behaviours (age 14)**	**0.4**	**0.03**	**< 0.001**		
	**Sex**	**0.15**	**0.03**	**< 0.001**		
	Ethnicity	0.004	0.01	0.79		
	**BMI**	**0.14**	**0.02**	**< 0.001**		
	Income	− 0.01	0.01	0.47		
Model 4	WASO	− 0.01	0.04	0.28	0.68	χ^2^(35, 5975) = 1230.38, CFI = 0.96, TLI = 0.95, RMSEA = 0.07, SRMR = 0.05
	**Restrictive Eating Behaviours (age 14)**	**0.4**	**0.03**	**< 0.001**		
	**Sex**	**0.15**	**0.03**	**< 0.001**		
	Ethnicity	0.004	0.01	0.78		
	**BMI**	**0.14**	**0.02**	**< 0.001**		
	Income	− 0.01	0.01	0.42		
Model 5	Social Jetlag	− 0.004	0.02	0.71	0.82	χ^2^(35, 5975) = 1223.56, CFI = 0.96, TLI = 0.94, RMSEA = 0.07, SRMR = 0.05
	**Restrictive Eating Behaviours (age 14)**	**0.4**	**0.03**	**< 0.001**		
	**Sex**	**0.15**	**0.03**	**< 0.001**		
	Ethnicity	0.004	0.01	0.77		
	**BMI**	**0.14**	**0.02**	**< 0.001**		
	Income	− 0.01	0.01	0.46		
Model 6	Wake-Up Time (School Day)	− 0.02	0.03	0.11	0.5	χ^2^(35, 5975) = 1214.05, CFI = 0.96, TLI = 0.94, RMSEA = 0.07, SRMR = 0.05
	**Restrictive Eating Behaviours (age 14)**	**0.4**	**0.03**	**< 0.001**		
	**Sex**	**0.15**	**0.03**	**< 0.001**		
	Ethnicity	0.003	0.01	0.81		
	**BMI**	**0.14**	**0.02**	**< 0.001**		
	Income	− 0.01	0.01	0.46		
Model 7	Wake-Up Time (School-Free Day)	− 0.02	0.03	0.06	0.5	χ^2^(35, 5975) = 1228.70, CFI = 0.96, TLI = 0.94, RMSEA = 0.07, SRMR = 0.05
	**Restrictive Eating Behaviours (age 14)**	**0.4**	**0.03**	**< 0.001**		
	**Sex**	**0.15**	**0.03**	**< 0.001**		
	Ethnicity	0.005	0.01	0.75		
	**BMI**	**0.14**	**0.02**	**< 0.001**		
	Income	− 0.01	0.01	0.39		
Model 8	Bedtime (School Day)	− 0.003	0.03	0.82	0.82	χ^2^(35, 5975) = 1223.03, CFI = 0.96, TLI = 0.94, RMSEA = 0.07, SRMR = 0.05
	**Restrictive Eating Behaviours (age 14)**	**0.4**	**0.03**	**< 0.001**		
	**Sex**	**0.15**	**0.03**	**< 0.001**		
	Ethnicity	0.004	0.01	0.79		
	**BMI**	**0.14**	**0.02**	**< 0.001**		
	Income	− 0.01	0.01	0.47		
Model 9	Bedtime (School-Free Day)	− 0.003	0.03	0.77	0.82	χ^2^(35, 5975) = 1214.97, CFI = 0.96, TLI = 0.95, RMSEA = 0.07, SRMR = 0.05
	**Restrictive Eating Behaviours (age 14)**	**0.4**	**0.03**	**< 0.001**		
	**Sex**	**0.15**	**0.03**	**< 0.001**		
	Ethnicity	0.004	0.01	0.78		
	**BMI**	**0.14**	**0.02**	**< 0.001**		
	Income	− 0.01	0.01	0.46		

*p-adjustment reported for all values relevant to hypothesis testing

**Table 3:** Overview SEM regression outcomes for time-use reported sleep indicators (Age 14) predicting restrictive eating behaviours (Age 17) (*N* = 2,164)

Model	Predictor	Beta	SE	*p*	*p* (adjusted)*	Fit Indices
Model 1	Sleep Duration (Week)	− 0.02	0.02	0.36	0.88	χ^2^(35, 2150) = 477.48, CFI = 0.96, TLI = 0.94, RMSEA = 0.07, SRMR = 0.05
	**Restrictive Eating Behaviours (age 14)**	**0.4**	**0.04**	**< 0.001**		
	**Sex**	**0.17**	**0.06**	**< 0.001**		
	Ethnicity	− 0.03	0.03	0.2		
	**BMI**	**0.12**	**0.04**	**< 0.001**		
	Income	− 0.04	0.02	0.12		
Model 2	Sleep Duration (Weekend)	0.02	0.02	0.32	0.88	χ^2^(35, 2150) = 479.55, CFI = 0.96, TLI = 0.94, RMSEA = 0.07, SRMR = 0.05
	**Restrictive Eating Behaviours (age 14)**	**0.4**	**0.04**	**< 0.001**		
	**Sex**	**0.17**	**0.06**	**< 0.001**		
	Ethnicity	− 0.03	0.03	0.21		
	**BMI**	**0.12**	**0.04**	**< 0.001**		
	Income	− 0.04	0.02	0.13		
Model 3	Daytime Napping (Week)	0.007	0.11	0.73	0.88	χ^2^(35, 2150) = 477.30, CFI = 0.96, TLI = 0.94, RMSEA = 0.07, SRMR = 0.05
	**Restrictive Eating Behaviours (age 14)**	**0.4**	**0.04**	**< 0.001**		
	**Sex**	**0.18**	**0.06**	**< 0.001**		
	Ethnicity	− 0.03	0.03	0.2		
	**BMI**	**0.12**	**0.04**	**< 0.001**		
	Income	− 0.04	0.02	0.13		
Model 4	Daytime Napping (Weekend)	− 0.003	0.1	0.88	0.88	χ^2^(35, 2150) = 477.47, CFI = 0.96, TLI = 0.94, RMSEA = 0.07, SRMR = 0.05
	**Restrictive Eating Behaviours (age 14)**	**0.4**	**0.04**	**< 0.001**		
	**Sex**	**0.18**	**0.06**	**< 0.001**		
	Ethnicity	− 0.03	0.03	0.2		
	**BMI**	**0.12**	**0.04**	**< 0.001**		
	Income	− 0.04	0.02	0.13		
Model 5	Night Awakening (Week)	0.008	0.12	0.69	0.88	χ^2^(35, 2150) = 477.25, CFI = 0.96, TLI = 0.94, RMSEA = 0.07, SRMR = 0.05
	**Restrictive Eating Behaviours (age 14)**	**0.4**	**0.04**	**< 0.001**		
	**Sex**	**0.18**	**0.06**	**< 0.001**		
	Ethnicity	− 0.03	0.03	0.2		
	**BMI**	**0.12**	**0.04**	**< 0.001**		
	Income	− 0.04	0.02	0.13		
Model 6	Night Awakening (Weekend)	− 0.007	0.11	0.71	0.88	χ^2^(35, 2150) = 476.26, CFI = 0.96, TLI = 0.94, RMSEA = 0.07, SRMR = 0.05
	**Restrictive Eating Behaviours (age 14)**	**0.4**	**0.04**	**< 0.001**		
	**Sex**	**0.18**	**0.06**	**< 0.001**		
	Ethnicity	− 0.03	0.03	0.2		
	**BMI**	**0.12**	**0.04**	**< 0.001**		
	Income	− 0.04	0.02	0.13		

*p-adjustment reported for all values relevant to hypothesis testing

**Table 4:** Overview SEM regression outcomes for restrictive eating behaviours (age 14) predicting overall sleep quality (Age 17) (*N* = 5,975)

Predictor	Beta	SE	*p*	Fit Indices
**Restrictive Eating Behaviours (age 14)**	**0.06**	**0.01**	**0.003**	χ^2^(28, 5975) = 218.87, CFI = 0.99, TLI = 0.99, RMSEA = 0.03, SRMR = 0.01
**Sleep Duration (School Day)**	**− 0.05**	**0.02**	**0.007**	
Sleep Duration (School-Free Day)	− 0.03	0.02	0.16	
**Sleep Latency**	**0.15**	**0.02**	**< 0.001**	
**WASO**	**0.11**	**0.03**	**< 0.001**	
Social Jetlag	0.02	0.02	0.33	
Wake-Up Time (School Day)	< 0.001	0.03	0.99	
Wake-Up Time (School-Free Day)	0.02	0.03	0.44	
Bedtime (School Day)	0.08	0.04	**< 0.001**	
**Bedtime (School-Free Day)**	0.003	0.04	0.88	
Sex	0.001	0.02	0.92	
Ethnicity	− 0.003	0.009	0.83	
BMI	− 0.003	0.02	0.84	
**Income**	**− 0.04**	**0.008**	**0.01**	

In contrast, restrictive eating behaviours at age 14 significantly predicted poor overall sleep quality at age 17, while controlling for all sleep indicators at baseline level (β = 0.06, SE = 0.01, *p* <.01). The model fit for this model was very good (χ^2^(28, 5975) = 218.87, CFI = 0.99, TLI = 0.99, RMSEA = 0.03, SRMR = 0.01), even though effect sizes were small.

### Mediation results

**Fig. 1:**
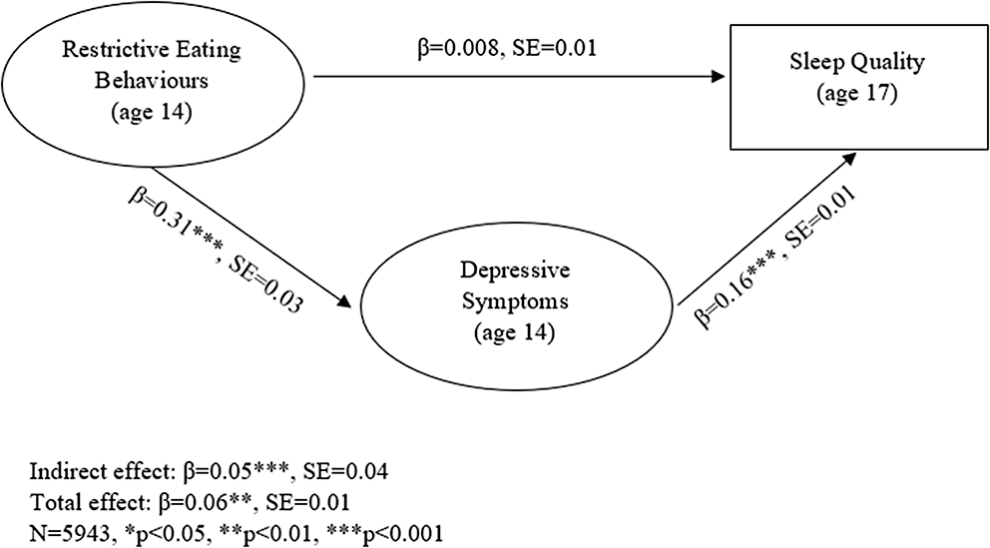
Mediation restrictive eating behaviours predicting overall sleep quality through depressive symptoms

Depressive symptoms fully mediated the positive association between restrictive eating behaviours and sleep quality (Fig. 1). The model fit for this model was very good (χ^2^(299, 5943) = 2926.64, CFI = 0.99, TLI = 0.98, RMSEA = 0.03, SRMR = 0.03).

## Discussion

This study explored the longitudinal relationship between restrictive eating behaviours and a variety of sleep measures within a large sample of UK adolescents. Significant associations between restrictive eating behaviours and a variety of sleep measures were identified within each timepoint even after controlling for key demographic characteristics. Significant prospective associations were identified between restrictive eating behaviours at age 14 and overall self-reported sleep quality at age 17, but not for the associations between individual sleep indicators at age 14 and subsequent restrictive eating behaviours at age 17. Even though all observed effects were small (standardised coefficients < 0.15), this is an important finding considering the longitudinal study design and large, general population sample.

Although this study did not find a bi-directional relationship between sleep and self-reported restrictive eating symptoms, further research in this area is necessary to address the methodological limitations of the MCS, in which sleep was not consistently assessed across time and restrictive eating assessments were not able to differentiate weight loss behaviours and intentions from clinically relevant disordered eating behaviours. As the concept of sleep quality encompasses a variety of sleep experiences [29], future research needs to further explore the combined effects of different sleep indicators on restrictive eating behaviours in adolescents. Moreover, while previous longitudinal research found positive associations between ED symptoms and sleep problems when investigating each directional effect individually [30, 31], there is a need to further explore the bi-directional relationship between restrictive disordered eating behaviours and sleep.

The significant positive association between restrictive eating behaviours and subsequent poor sleep quality extends previous findings on restrictive disordered eating in young adults [30], and university students [32]. Thereby, it is important to highlight that the MCS only provides information on general dieting behaviours and weight loss intentions that were very common within our sample. While this assessment does not reflect clinical cut-offs for disordered eating, these high numbers are in line with findings from other large longitudinal studies, which found about half of adolescent girls to report past-year dieting for weight loss as well as *unhealthy* weight loss behaviours (e.g., fasting, skipping meals, substituting food) [33]. With dieting in adolescence being considered a risk factor for the development of disordered eating [e.g., 6], current findings should be extended to assess how a variety of dieting behaviours and cognitions relate to sleep difficulties and behaviours.

While previous research controlled for depressive symptoms to examine the relationship between sleep and disordered eating, the present study identified depressive symptoms as a potential mediator for the relationship between restrictive eating behaviours and sleep. Yet, as the current study assessed depressive symptoms and restrictive eating behaviours at the same time (age 14), future research is needed to disentangle the temporal association between restrictive eating behaviours, depressive symptoms, and sleep. Furthermore, considering the high prevalence rates of disordered eating in young people (22.35%) [34], as well as unique biopsychological risks during this developmental stage [35], it is likely that some of the young people who endorsed restrictive eating behaviours in the current sample were also experiencing disordered eating. This might have impacted study findings, as across both cross-sectional and longitudinal studies, ED symptomatology has been shown to be moderately positively associated with rumination [36] and depressive symptoms [37].

Meanwhile, a reciprocal relationship between sleep and mood disturbances is reflected within the diagnostic criteria for mood disorders, which includes several sleep problems as part of the core criteria, with insomnia and hypersomnia being considered risk factors for the development and recurrence of these disorders [38]. Thus, more longitudinal research is needed to investigate causal relationships between restrictive eating behaviours, depressive symptoms and sleep, to investigate if depressive symptoms constitute a true mediating mechanism or if depressive symptoms act as a confounder in this relationship. Current findings are only a first step in evaluating this relationship longitudinally, suggesting that weight loss-focused behaviours may lead to poorer sleep quality through the presence of depressive symptoms. Thus, targeting mood in the context of restrictive eating may limit negative impact of restrictive eating behaviours on sleep quality, potentially addressing tertiary prevention goals in EDs [39].

The use of a variety of sleep indicators provided the opportunity to test individual effects of sleep characteristics, sleep disturbances, and global assessments of perceived sleep quality. Previous research has identified intra-individual sleep variability as the most meaningful predictor of mental health difficulties [40]. Even though sleep indicators in the present study were not significantly associated with increases in restrictive eating behaviours between ages 14 and 17, significant concurrent associations suggest that sleep may be a notable marker of restrictive eating behaviours in adolescence. This included not only sleep difficulties but also self-reported typical sleep behaviours. Future longitudinal studies should make use of repeated measures of validated scales measuring sleep difficulties as well as general sleep behaviours to further investigate the role of different sleep dimensions in the development and progression of disordered eating behaviours.

Finally, despite several limitations of the diary assessments (e.g., the specified timeframe, broad answer categories, lack of consideration for holidays), it was surprising that no significant associations were found between TUD sleep indicators and restrictive eating behaviours. Similar to findings of the present study, self-reported sleep quality has previously been found to be the best predictor for individuals’ mental and physical health [41], highlighting the importance of choosing the most meaningful sleep indicators in mental health research. Therefore, future studies may benefit from including a variety of sleep measurements (e.g., self-reports, ecological momentary assessments, actigraphy) to assess the relationship between sleep and disordered eating to capture different sleep related experiences [42].

### Strengths and limitations

The present study adds to the current literature by providing insights into the longitudinal relationship between sleep and restrictive eating behaviours within a large adolescent sample. Although this study provided the opportunity to investigate bi-directional associations, the MCS does not include repeated assessments of the same sleep characteristics (individual sleep indicators vs. a one-item sleep quality assessment) and methods (i.e., diary assessment), limiting the comparability of directional effects. Future cross-lagged study designs are needed to account for construct-specific changes across time, ideally considering both inter-individual as well as intra-individual changes. For example, the present study did not consider any developmental trajectories of sleep across the two assessment points, a time during which adolescents’ circadian timing system undergoes a phase delay due to psychosocial and bioregulatory changes [43], resulting in a heightened probability for ill-timed and inadequate sleep [44]. Thus, the assessment of longitudinal bi-directional assessments needs to consider developmental sleep changes that can occur independently of restrictive eating behaviours.

Moreover, even though single items utilised in the present study resemble validated assessments of sleep (i.e., the Athens Insomnia Scale), current findings need to be replicated using validated measures of sleep routines, difficulties, and overall sleep quality. Categorical measurements of sleep duration limit the exploration of more subtle variations in sleep patterns. Furthermore, a considerable limitation of the TUD information provided is the assessment of sleep over 24 h from 4am to 4am (i.e., not one continuous night of sleep). Thus, current findings need to be interpreted in consideration of these methodological limitations.

Importantly, the present study only included general restrictive eating behaviours, as the MCS does not include a measure for disordered eating behaviours in adolescence. Therefore, present findings are limited in their generalisability regarding ED risk factors beyond common experiences of dieting and weight loss intentions. Further research into a variety of disordered eating behaviours, especially binge-purge subtype-specific behaviours [14], are needed, considering that previous research has clearly established concurrent associations between ED symptomatology and sleep problems within clinical ED populations [15], as well as links between sleep behaviours and loss-of-control eating [40].

## Conclusion

Findings from this study offer valuable insights into the longitudinal relationship between sleep and restrictive eating behaviours, including the influential role of depressive symptoms. Methodological challenges for the assessment of sleep patterns and disturbances are discussed, recommending the inclusion of a variety of sleep measures for research and diagnostics.

## Electronic supplementary material

Below is the link to the electronic supplementary material.

Supplementary Material 1

## Data Availability

The data utilised for this project can be accessed through the UK data service (https://ukdataservice.ac.uk/)/. The analysis code for this study is publicly available at https://github.com/M-COpitz/MCS_Sleep_Disordered_Eating.git.
