# Supplementary material for: Investigating the longitudinal bi-directional relationship between self-reported restrictive eating behaviours and sleep in UK adolescents within the Millennium Cohort Study
Source: Eur Child Adolesc Psychiatry. 2025 Jan 28;34(8):2405–16. doi: 10.1007/s00787-025-02641-9 (PMC12397170; doi:10.1007/s00787-025-02641-9)
Supplement: Supplementary file 1 — Supplementary Material 1 [file 787_2025_2641_MOESM1_ESM.docx]

Supplementary Material: Investigating the Longitudinal Bi-Directional Relationship between Self-Reported Restrictive Eating Behaviours and Sleep in UK Adolescents within the Millennium Cohort Study (European Child & Adolescent Psychiatry)

Marie-Christine Opitz^1^*, Giulia Gaggioni^2^, Nora Trompeter^3^, Francisco Diego Rabelo da Ponte^4^, Sylvane Desrivières^4^, Nadia Micali^3,5^, Ulrike Schmidt^6, 7^, Helen Sharpe^1^

^1^Department of Clinical Psychology, School of Health in Social Sciences, University of Edinburgh, Scotland

^2^Division of Psychiatry, Centre for Clinical Brain Sciences, University of Edinburgh, Scotland

^3^Great Ormond Street Institute of Child Health, University College London, UK

^4^Social Genetic and Developmental Psychiatry Centre, Institute of Psychiatry, Psychology and Neuroscience, King’s College London, UK

^5^Ballerup Psychiatric Centre, Eating Disorder Research Unit, Copenhagen, Denmark

^6^Centre for Research in Eating and Weight Disorders, Institute of Psychiatry, Psychology and Neuroscience, King’s College London, UK

^7^South London and Maudsley NHS Foundation Trust, London, UK

*corresponding author: mopitz@ed.ac.uk

Supplementary Table 1. Available Relevant Participant Information for MCS Sweep 6 and Sweep 7

| **Explored Availability** | **N Participants** |
| --- | --- |
| Issued at Sweep 6 (14 years) | 15,414 |
| Issued at Sweep 7 (17 years) | 14,438 |
| Completed all Restrictive Eating Measures at Sweep 6 (14 years) | 11,087 |
| Completed all Restrictive Eating Measures at Sweep 7 (17 years) | 6,681^[[1]](#footnote-1)^ |
| Completed all Restrictive Eating Measures at both Sweep 6 (age 14) And Sweep 7 (age 17) | 6,180 |
| Completed Week Bed Time (age 14) | 11,322 |
| Completed Week Wake-Up Time (age 14) | 11,333 |
| Completed Weekend Bed Time (age 14) | 11,324 |
| Completed Weekend Wake-Up Time (age 14) | 11,321 |
| Completed Sleep Onset Latency (age 14) | 11,263 |
| Completed Wake After Sleep Onset (age 14) | 11,299 |
| Completed Item on Overall Sleep Quality (age 17) | 6,557 |
| Completed Item on Overall Sleep Quality (age 17) and all Restrictive Eating Measures at both Sweep 6 (age 14) And Sweep 7 (age 17) | 6,060 |
| Completed all Items on the Short Moods and Feeling Questionnaire (SMFQ, Symptoms of Depression) | 11,042^[[2]](#footnote-2)^ |
| Completed all Sleep Variables (age 14 and 17) and all Restrictive Eating Measures at both Sweep 6 (age 14) And Sweep 7 (age 17) | 6,027 |
| Completed all Sleep Variables (age 14 and 17) and all Restrictive Eating Measures at both Sweep 6 (age 14) And Sweep 7 (age 17) and relevant covariates (sex, ethnicity, BMI category, parent-reported household income) | **5,832^[[3]](#footnote-3)^** |
| **Note**: This table refers to the initial 4 items of restrictive eating behaviours |  |

R Packages Used

Descriptive statistics were investigated using the R packages “psych” version 2.4.1 (Revelle & Revelle, 2015), “lessR” version 4.3.0 (Gerbing et al., 2023), and “tidyverse” version 2.0.0 (Wickham et al., 2019). To specify the latent variable model, the R package “lavaan” version 0.6.16 (Rosseel, 2012) was used. To impute missing values “MICE” version 3.16.0 (van Buuren & Groothuis-Oudshoorn, 2011) was utilised. All R packages used to implement the analysis code, can be found at GitHub: <https://github.com/M-COpitz/MCS_Sleep_Disordered_Eating.git>.

Additional Information on Utilised Measures from the Millennium Cohort Study

Restrictive eating behaviours was operationalized as a multiple indicator latent variable at both time points, which allowed for the evaluation of the latent factor model fit and factor structure. In a protocol deviation, the item ‘overweight perception’ was not included as part of the restrictive eating factor, due to comparatively low (<.30) factor loadings at both time points and its conceptual ambiguity. Even though the final three indicators are not representative of the full spectrum of disordered eating behaviours (e.g., loss-of-control eating), they resemble measures utilised in other cohort research studies to assess dietary restraint and weight concerns as ED risk factors (cf. Evans et al., 2017).

Sleep was assessed using multiple indicators (Supplementary Table 2). At age 14, cohort members were asked about their typical bed and wake times during a school day and a day they did not have to attend school. This information was used to create the following variables: typical sleep duration, wake-up and bedtimes, and social jetlag. To calculate these variables for school days and school-free days, each answer category was transformed into one number (e.g., 20.5 for “Before 9pm”, 8.5 for “08:00-08:59”), and wake-up times subtracted from bed times (see Collings (2021)). To calculate social jetlag, midpoints of sleep durations were identified for both school days and school-free days and time differences used to illustrate the amount of social jetlag. Positive values indicate later sleep timing on school-free nights and negative values indicate later sleep timing on school nights. For time-use sleep assessments, participants were asked to record their activities from 4am on the first day until 4am the second day (e.g., “Sleeping and resting (including sick in bed)”).

Sex, ethnicity, weight category, and household income were utilised as provided by the MCS derived datasets.

| Supplementary Table 2. Sleep Variables Included in the Millennium Cohort Study at Age 14 |  |
| --- | --- |
| **Sleep Variable** | **Response Options** |
| About what time do you usually go to sleep on a school night? | “Before 9pm”  “9:00-9:59pm”  “10:00-10:59pm”  “11:00-midnight”  “After midnight” |
| About what time do you usually wake up in the morning on a school day? | “Before 6am”  “6:00-6:59am”  “7:00-7:59am”  “8:00-8:59am”  “After 9am” |
| About what time do you usually go to sleep on the nights when you do not have school the next day? | “Before 9pm”  “9:00-9:59pm”  “10:00-10:59pm”  “11:00-midnight”  “After midnight” |
| About what time do you wake up in the morning on the days when you do not have school? | “Before 8am”  “8:00-8:59am”  “9:00-9:59am”  “10:00-10:59am”  “11:00-11:59am” |
| During the last four weeks, how long did it usually take for you to fall asleep? | “0-15min”  “16-30min”  “31-45min”  “46-60min”  “More than 60min” |
| During the last four weeks, how often did you awaken during your sleep time and have trouble falling back to sleep again? | “All of the time”  “Most of the time”  “A good bit of the time”  “Some of the time”  “A little of the time”  “None of the time” |
|  | |

Survey Weights and Missing Data in the Current Study

Although survey weights are available for the MCS cohort, R currently has no package available that allows for the adjustment of survey weights within structural equation modelling (SEM). Thus, survey weighting could not be implemented in these analyses, limiting the generalisability of study findings. An inspection of missing data for all restricive eating variables did not reveal any specific pattern of missingness for individual items. Little’s MAR test was non-significant for both time points, indicating restricive eating variables were missing at random. Ethnicity values were missing for n=3 participants (across waves), who were subsequently classified as “other ethnicities” in all analyses. Missing data for BMI were identified for n=66 (0.01%, analytic sample) and n=14 participants (0.007%, subsample). Multiple Imputation by Chained Equation (MICE) was implemented to impute missing values for these participants. However, current R packages cannot account for WLSMV estimators when pooling imputed data during the estimation of SEM models. As our first priority was to use the most appropriate estimator for all analyses, and missing data were low (≤.01%), relevant analyses were conducted on participants with full BMI data (N=5,975 and N=2,150) as well as on the specified five imputed datasets individually. Imputations did not significantly differ between the imputed data sets and regression findings did not differ across the complete data sets. Thus, we only report findings from the complete datasets where relevant.

Longitudinal Measurement Invariance for the Latent Restrictive Eating Factor

In order to assess model fit, the present study followed the recommendations by Chen (2007) who suggests a change of .01 in CFI, paired with changes in RMSEA of .015 and SRMR of .030 (for metric invariance) or .015 (for scalar or residual invariance) as cut-off values for an acceptable fit. Model fit was good for all specified models, with the best fit for metric invariance, restricting item factor loadings to be the same across measurements (Isiordia & Ferrer, 2018). Thus, the three restrictive eating items represent the latent construct consistently at both data collection points (Mackinnon et al., 2022). Relevant model fit outcomes are presented in Supplementary Table 3. The SEM model showed a very good fit (factor loadings ≥.69), indicating a unidimensional latent factor of restrictive-type restrictive eating.

Supplementary Table 3. Longitudinal Measurement Invariance

| **Type of Measurement Invariance** | **CFI** | **RMSEA** | **SRMR** | **ΔCFI** | **ΔRMSEA** | **ΔSRMR** |
| --- | --- | --- | --- | --- | --- | --- |
| **Configural** | 1.00 | .020 | .010 | - | - | - |
| **Metric** | .999 | .018 | .012 | .001* | .002* | .002* |
| **Scalar** | .994 | .045 | .029 | .005 | .027 | .017 |
| **Residual** | .992 | .047 | .030 | .002 | .002 | .001 |
| N=6,041 | | | | | | |

| Supplementary Table 4. Affirmative Replies for Restricive Eating Indicators by Sex, BMI Categories, Household Income Quintiles, and Ethnicity | | | | |
| --- | --- | --- | --- | --- |
|  | **Reported by Sex** | **Reported by UK90 BMI Category** | **Reported by Household Income Quintile** | **Reported by Ethnicity** |
| **Weight Loss Intention (age 14)** | 30.6% of Boys  51.3% of Girls | 2.1% of “underweight”  26.5% of “healthy weight”  60% of “overweight”  79% of “obese” | 51.3% of “lower quantile”  47.9% of “second quantile”  41.1% of “third quantile”  42.4% of “fourth quantile”  36.5% of “highest quantile” | 41.3% of “White”  42.9% of “Mixed Ethnicity”  44.4% of “Indian”  49.7% of “Pakistani and Bangladeshi”  47.2% of “Black or Black British”  48.2% of “Other Ethnic Group (incl. Chinese” |
| **Dietary Restriction (age 14)** | 33.5% of Boys  53.4% of Girls | 12.8% of “underweight”  32.9% of “healthy weight”  57.9% of “overweight”  71.5% of “obese” | 50.8% of “lower quantile”  46.2% of “second quantile”  44.8% of “third quantile”  45.5% of “fourth quantile”  40.7% of “highest quantile” | 43.9% of “White”  47.7% of “Mixed Ethnicity”  42.7% of “Indian”  47.8% of “Pakistani and Bangladeshi”  55.1% of “Black or Black British”  51.1% of “Other Ethnic Group (incl. Chinese” |
| **Excessive Exercise (age 14)** | 53.4% of Boys  64.8% of Girls | 19.2% of “underweight”  49.7% of “healthy weight”  74% of “overweight”  83.1% of “obese” | 61.6% of “lower quantile”  62% of “second quantile”  61.1% of “third quantile”  61.4% of “fourth quantile”  55.8% of “highest quantile” | 59.4% of “White”  59% of “Mixed Ethnicity”  57.9% of “Indian”  63.3% of “Pakistani and Bangladeshi”  69.3% of “Black or Black British”  59.1% of “Other Ethnic Group (incl. Chinese” |
| **Weight Loss Intention (age 17)** | 31.4% of Boys  61% of Girls | 11.7% of “underweight”  34.4% of “healthy weight”  64.7% of “overweight”  79.9% of “obese” | 50.6% of “lower quantile”  51% of “second quantile”  49.7% of “third quantile”  47.5% of “fourth quantile”  45% of “highest quantile” | 47.6% of “White”  45.9% of “Mixed Ethnicity”  46.2% of “Indian”  52.5% of “Pakistani and Bangladeshi”  51.7% of “Black or Black British”  54% of “Other Ethnic Group (incl. Chinese” |
| **Dietary Restriction (age 17)** | 37.7% of Boys  63.6% of Girls | 19.3% of “underweight”  42% of “healthy weight”  64.4% of “overweight”  76.4% of “obese” | 50.6% of “lower quantile”  53% of “second quantile”  53% of “third quantile”  52.5% of “fourth quantile”  51.9% of “highest quantile” | 52.3% of “White”  54.9% of “Mixed Ethnicity”  51.5% of “Indian”  49.7% of “Pakistani and Bangladeshi”  51.7% of “Black or Black British”  56.2% of “Other Ethnic Group (incl. Chinese” |
| **Excessive Exercise (age 17)** | 53.7% of Boys  69.7% of Girls | 22.3% of “underweight”  54.3% of “healthy weight”  75% of “overweight”  83% of “obese” | 58.9% of “lower quantile”  63.2% of “second quantile”  63.4% of “third quantile”  64.8% of “fourth quantile”  61.9% of “highest quantile” | 63.2% of “White”  58.3% of “Mixed Ethnicity”  59.1% of “Indian”  59.7% of “Pakistani and Bangladeshi”  63.6% of “Black or Black British”  67.9% of “Other Ethnic Group (incl. Chinese” |
| **Note:** This table is based on the complete sex, BMI, ethnicity, and income data | | | | |

Supplementary Table 5. Associations Between Sleep Variables and Restrictive Eating Variables (N=6,041)

|  | Sleep Duration (Week) | Sleep Duration (Weekend) | Sleep Onset Latency | WASO | Social Jetlag | Overall Sleep Quality (age 17) | Weight Loss Intention (age 14) | Dietary Restriction (age 14) | Excessive Exercise (age 14) | Weight Loss Intention (age 17) | Dietary Restriction (age 17) | Excessive Exercise (age 17) |
| --- | --- | --- | --- | --- | --- | --- | --- | --- | --- | --- | --- | --- |
| Sleep Duration (Week) | 1 |  |  |  |  |  |  |  |  |  |  |  |
| Sleep Duration (Weekend) | .13***  [.11; .14] | 1 |  |  |  |  |  |  |  |  |  |  |
| Sleep Onset Latency | .10***  [.08; .13] | .07***  [.05; .10] | 1 |  |  |  |  |  |  |  |  |  |
| WASO | .10***  [.08; .13] | .09***  [.06; .11] | .25***  [.22; .27] | 1 |  |  |  |  |  |  |  |  |
| Social Jetlag | -.10***  [-.13; -.08] | .44*** [.42; .46] | .06***  [.04; .09] | .05***  [.02; .07] | 1 |  |  |  |  |  |  |  |
| Overall Sleep Quality (age 17) | .09***  [.07; .10] | .05***  [.03; .06] | .21***  [.18; .23] | .18***  [.16; .21] | .05***  [.03; .08] | 1 |  |  |  |  |  |  |
| Weight Loss Intention (age 14) | .13***  [.11; .16] | .06***  [.04; .08] | .05***  [.03; .08] | .14***  [.12; .16] | .09***  [.07; .12] | .10***  [.07; .12] | 1 |  |  |  |  |  |
| Dietary Restriction (age 14) | .15***  [.12; .17] | .09***  [.06; .11] | .08***  [.05; .10] | .16***  [.14; .18] | .07***  [.05; .10] | .11***  [.09; .14] | .55***  [.53; .57] | 1 |  |  |  |  |
| Excessive Exercise (age 14) | .08***  [.06; .11] | .04  [.00; .06] | .03**  [.01; .06] | .11***  [.09; .14] | .08***  [.05; .10] | .05**  [.03; .07] | .51***  [.49; .53] | .52***  [.50; .54] | 1 |  |  |  |
| Weight Loss Intention (age 17) | .09***  [.06; .11] | .02  [0.00; 0.05] | .03*  [.008; .06] | .11***  [.09; .14] | .09***  [.06; .11] | .12***  [.10; .15] | .47***  [.45; .49] | .36***  [.34; .38] | .33***  [.31; .35] | 1 |  |  |
| Dietary Restriction (age 17) | .10***  [.07; .12] | .05**  [.03; .08] | .04***  [.02; .07] | .08***  [.05; .10] | .04** [.01; .06] | .11***  [.09; .14] | .38***  [.36; .40] | .38***  [.36; .40] | .33***  [.31; .35] | .54***  [.52; .56] | 1 |  |
| Excessive Exercise (age 17) | .06***  [.04; .08] | .05**  [.03; .08] | .01  [-.01; .04] | .03*  [.01; .06] | .02  [-.01; .05] | .04* [.00; .07] | .34***  [.32; .36] | .30***  [.28; .32] | .32***  [.30; .34] | .49***  [.47; .50] | .55***  [.53; .57] | 1 |
| *p<.05, **p<.01, ***p<.001; correlations between dichotomous variables are based on phi coefficients; all other correlations were calculated using Cramer’s V (possible values 0 to 1), except for correlations with social jetlag, which were based on point-biserial correlation coefficients with possible values -1 to 1; significance testing was conducted using chi-square tests, p-values were adjusted for multiple testing using the Benjamini-Hochberg correction | | | | | | | | | | | | |

Supplementary Table 6. Associations Between Wake-Up and Bed-Times and Other Self-Reported Variables (N=6,041)

|  | Sleep Duration (Week) | Sleep Duration (Weekend) | Sleep Onset Latency | WASO | Social Jetlag | Overall Sleep Quality (age 17) | Weight Loss Intention (age 14) | Dietary Restriction (age 14) | Excessive Exercise (age 14) | Weight Loss Intention (age 17) | Dietary Restriction (age 17) | Excessive Exercise (age 17) |
| --- | --- | --- | --- | --- | --- | --- | --- | --- | --- | --- | --- | --- |
| Wake-Up Time (Week) | .39***  [.37; .42] | .15***  [.13; .18] | -.01  [-.03; .02] | -.07***  [-.09; -.04] | -.18***  [-.21; -.16] | .03  [.00; .05] | -.06***  [-.08; -.03] | -.06***  [-.09; -.03] | -.05***  [-.07; -.02] | -.08***  [-.10; -.05] | -.05***  [-.08; -.03] | -.05***  [-.07; -.02] |
| Wake-Up Time (Weekend) | .12***  [.09; .14] | .60***  [.58; .63] | .08***  [.06; .11] | .03  [.001; .05] | .66***  [.64; .67] | .06***  [.04; .09] | .04**  [.02; .07] | .03  [.001; .05] | .03*  [.004; .05] | .04**  [.01; .06] | -.01  [-.03; .02] | -.03*  [-.06; -.01] |
| Bedtime (Week) | .62***  [.60; .65] | .16***  [.13; .18] | .16***  [.14; .18] | .09***  [.07; .12] | -.04**  [-.06; -.01] | .16***  [.14; .19] | .11***  [.09; .13] | .13***  [.11; .15] | .06***  [.03; .08] | .07***  [.04; .09] | .06***  [.04; .09] | .03**  [.01; .06] |
| Bedtime (Weekend) | .38***  [.36; .41] | .18***  [.15; .20] | .14***  [.12; .16] | .10***  [.08; .13] | .40***  [.37; .42] | .13***  [.11; .16] | .12***  [.10; .15] | .13***  [.11; .15] | .08***  [.05; .10] | .07***  [.05; .10] | .06***  [.03; .08] | .04**  [.01; .06] |

*p<.05, **p<.01, ***p<.001; correlations between dichotomous variables are based on phi coefficients; all other correlations were calculated using Cramer’s V (possible values 0 to 1), except for correlations with social jetlag, which were based on point-biserial correlation coefficients with possible values -1 to 1; significance testing was conducted using chi-square tests, p-values were adjusted for multiple testing using the Benjamini-Hochberg correction

Supplementary Table 7. Associations Between Sleep Variables (N=2,164)

|  | Sleep Duration (Week) | Sleep Duration (Weekend) | Sleep Onset Latency | WASO | Social Jetlag | Overall Sleep Quality (age 17) | Wake-Up Time (Week) | Wake-Up Time (Weekend) | Bedtime (Week) | Bedtime (Weekend) |
| --- | --- | --- | --- | --- | --- | --- | --- | --- | --- | --- |
| TUD Sleep Duration (Week) | .19***  [.17; .22] | .09***  [.07; .11] | .02  [.00; .06] | .03  [.00; .07] | .002  [-.04; .04] | .07**  [.05; .09] | .15***  [.11; .19] | .09**  [.05; .13] | .23***  [.19; .28] | .18***  [.14; .22] |
| TUD Sleep Duration (Weekend) | .06**  [.04; .09] | .12***  [.10; .16] | .04  [.00; .08] | .05  [.00; .09] | .09***  [.05; .13] | .05  [.00; .07] | .06  [.00; .11] | .13***  [.10; .18] | .08*  [.05; .13] | .07  [.04; .11] |
| TUD Daytime Napping (Week) | .05  [.00; .10] | .04  [.00; .08] | .003  [-.02; .03] | .04  [.02; .07] | .03  [-.01; .07] | .02  [.00; .06] | .02  [-.005; .05] | .02  [-.005; .05] | .05  [-.005; .05] | .03  [-.005; .05] |
| TUD Daytime Napping (Weekend) | .02  [.00; .06] | .04  [.00; .08] | .01  [-.02; .04] | .02  [-.01; .04] | -.001  [-.04; .04] | .04  [.00; .08] | -.04  [-.07; -.02] | -.02  [-.05; .005] | .00  [-.03; .03] | .02  [-.005; .05] |
| TUD Night Time Awakening (Week) | .06  [.00; .10] | .03  [.00; .08] | .02  [-.01; .04] | .04  [.01; .06] | .01  [-.03; .05] | .02  [.00; .06] | -.01  [-.05; .005] | -.01  [-.05; .005] | .01  [-.02; .04] | .01  [-.02; .04] |
| TUD Night Time Awakening (Weekend) | .03  [.00; .07] | .04  [.00; .08] | .02  [-.01; .04] | .03 [.003; .05] | .03  [-.02; .07] | .03  [.00; .07] | -.03  [-.02; .04] | -.01  [-.02; .04] | -.02  [-.05; .005] | .00  [-.03; .03] |

TUD=Time Use Data, *p<.05, **p<.01, ***p<.001; correlations between dichotomous variables are based on phi coefficients; all other correlations were calculated using Cramer’s V (possible values 0 to 1), except for correlations with social jetlag, which were based on point-biserial correlation coefficients with possible values -1 to 1; significance testing was conducted using chi-square tests, p-values were adjusted for multiple testing using the Benjamini-Hochberg correction

Supplementary Table 8. Associations Between Time Use Sleep Variables (N=2,164)

|  | TUD Sleep Duration (Week) | TUD Sleep Duration (Weekend) | TUD Daytime Napping (Week) | TUD Daytime Napping (Weekend) | TUD Night Time Awakening (Week) | TUD Night Time Awakening (Weekend) |
| --- | --- | --- | --- | --- | --- | --- |
| TUD Sleep Duration (Week) | 1 |  |  |  |  |  |
| TUD Sleep Duration (Weekend) | .11*  [.09; .13] | 1 |  |  |  |  |
| TUD Daytime Napping (Week) | .24***  [.20; .28] | .04  [.00; .08] | 1 |  |  |  |
| TUD Daytime Napping (Weekend) | .07*  [.04; .12] | .13***  [.09; .17] | .14***  [.12; .16] | 1 |  |  |
| TUD Night Time Awakening (Week) | .14***  [.10; .18] | .04  [.00; .08] | .14***  [.12; .17] | .05  [.03; .08] | 1 |  |
| TUD Night Time Awakening (Weekend) | .04  [.00; .08] | .17***  [.13; .22] | .08**  [.05; .10] | .06  [.03; .08] | .09***  [.07; .12] | 1 |

TUD=Time Use Data, *p<.05, **p<.01, ***p<.001; correlations between dichotomous variables are based on phi coefficients; all other correlations were calculated using Cramer’s V (possible values 0 to 1), except for correlations with social jetlag, which were based on point-biserial correlation coefficients with possible values -1 to 1; significance testing was conducted using chi-square tests, p-values were adjusted for multiple testing using the Benjamini-Hochberg correction

Supplementary Table 9. Associations Between Time Use Diary Sleep Variables and Restrictive Eating Variables (N=2,164)

|  | Weight Loss Intention (age 14) | Dietary Restriction (age 14) | Excessive Exercise (age 14) | Weight Loss Intention (age 17) | Dietary Restriction (age 17) | Excessive Exercise (age 17) |
| --- | --- | --- | --- | --- | --- | --- |
| TUD Sleep Duration (Week) | .07  [.00; .10] | .05  [.00; .10] | .03  [.00; .07] | .05  [.00; .09] | .07  [.04; .11] | .05  [.00; .09] |
| TUD Sleep Duration (Weekend) | .05  [.00; 09] | .03  [.00; .07] | .05  [.00; .09] | .07  [.00; .11] | .03  [.00; .07] | .02  [.00; .06] |
| TUD Daytime Napping (Week) | .05  [.02; .07] | .07**  [.05; .10] | .02  [-.003; .05] | .04  [.02; .07] | .05  [.02; .07] | .03  [-.0002; .05] |
| TUD Daytime Napping (Weekend) | .03  [.003; .05] | .004  [-.02; .03] | .003  [-.02; .03] | .04  [.01; .06] | -.01  [-.03; .02] | .002  [-.02; .03] |
| TUD Night Time Awakening (Week) | .01  [-.02; .03] | .05  [.02; .07] | .04  [.01; .06] | .03  [.01; .06] | .05  [.03; .08] | .01  [-.01; .04] |
| TUD Night Time Awakening (Weekend) | .02  [-.01; .04] | .05  [.02; .07] | .01  [-.01; .04] | .01  [-.02; .04] | .01  [-.02; .04] | .01  [-.01; .04] |

TUD=Time Use Data, *p<.05, **p<.01, ***p<.001; correlations between dichotomous variables are based on phi coefficients; all other correlations were calculated using Cramer’s V (possible values 0 to 1); significance testing was conducted using chi-square tests, p-values were adjusted for multiple testing using the Benjamini-Hochberg correction

Supplementary Table 10: Cross-Sectional Regression Outcomes for Self-Reported Sleep Characteristics and Restrictive Eating (N=5,975)

| Model | Predictor | Beta | SE | p | p (adjusted)* | Fit Indices |
| --- | --- | --- | --- | --- | --- | --- |
| Model 1 | **Sleep Duration (Week)** | **-.12** | **.02** | **<.001** | **<.001** | χ^2^(10, 5975)=160.19, CFI=.99, TLI=.99, RMSEA=.04, SRMR=.02 |
|  | **Sex** | **.22** | **.03** | **<.001** |  |  |
|  | Ethnicity | .03 | .01 | .04 |  |  |
|  | **BMI** | **.49** | **.02** | **<.001** |  |  |
|  | Income | -.003 | .01 | .84 |  |  |
| Model 2 | **Sleep Duration (Weekend)** | **-.08** | **.02** | **<.001** | **<.001** | χ^2^(10, 5975)=163.10, CFI=.99, TLI=.98, RMSEA=.04, SRMR=.02 |
|  | **Sex** | **.23** | **.03** | **<.001** |  |  |
|  | Ethnicity | .02 | .01 | .06 |  |  |
|  | **BMI** | **.49** | **.02** | **<.001** |  |  |
|  | Income | -.009 | .01 | .49 |  |  |
| Model 3 | **Sleep Onset Latency** | **.06** | **.03** | **<.001** | **<.001** | χ^2^(10, 5975)=159.35, CFI=.99, TLI=.99, RMSEA=.04, SRMR=.02 |
|  | **Sex** | **.22** | **.03** | **<.001** |  |  |
|  | Ethnicity | .03 | .01 | .06 |  |  |
|  | **BMI** | **.49** | **.02** | **<.001** |  |  |
|  | Income | -.005 | .01 | .73 |  |  |
| Model 4 | **WASO** | **.13** | **.04** | **<.001** | **<.001** | χ^2^(10, 5975)=159.63, CFI=.99, TLI=.99, RMSEA=.04, SRMR=.02 |
|  | **Sex** | **.21** | **.03** | **<.001** |  |  |
|  | Ethnicity | .02 | .01 | .07 |  |  |
|  | **BMI** | **.49** | **.02** | **<.001** |  |  |
|  | Income | .009 | .01 | .53 |  |  |
| Model 5 | **Social Jetlag** | **.06** | **.02** | **<.001** | **<.001** | χ^2^(10, 5975)=149.67, CFI=.99, TLI=.99, RMSEA=.03, SRMR=.02 |
|  | **Sex** | **.22** | **.03** | **<.001** |  |  |
|  | Ethnicity | .02 | .01 | .15 |  |  |
|  | **BMI** | **.49** | **.02** | **<.001** |  |  |
|  | Income | .001 | .01 | .95 |  |  |
| Model 6 | **Wake-Up Time (Week)** | **-.04** | **.03** | **.001** | **.001** | χ^2^(10, 5975)=149.18, CFI=.99, TLI=.99, RMSEA=.03, SRMR=.02 |
|  | **Sex** | **.22** | **.03** | **<.001** |  |  |
|  | Ethnicity | .02 | .01 | .12 |  |  |
|  | **BMI** | **.49** | **.02** | **<.001** |  |  |
|  | Income | -.008 | .01 | .56 |  |  |
| Model 7 | Wake-Up Time (Weekend) | .02 | .03 | .17 | .17 | χ^2^(10, 5975)=149.53, CFI=.99, TLI=.99, RMSEA=.03, SRMR=.02 |
|  | **Sex** | **.22** | **.03** | **<.001** |  |  |
|  | Ethnicity | .02 | .01 | .10 |  |  |
|  | **BMI** | **.49** | **.02** | **<.001** |  |  |
|  | Income | -.004 | .01 | .75 |  |  |
| Model 8 | **Bed Time (Week)** | **.10** | **.04** | **<.001** | **<.001** | χ^2^(10, 5975)=171,89, CFI=.99, TLI=.98, RMSEA=.04, SRMR=.02 |
|  | **Sex** | **.22** | **.03** | **<.001** |  |  |
|  | Ethnicity | .03 | .01 | .04 |  |  |
|  | **BMI** | **.49** | **.02** | **<.001** |  |  |
|  | Income | .003 | .01 | .84 |  |  |
| Model 9 | **Bed Time (Weekend)** | **.11** | **.04** | **<.001** | **<.001** | χ^2^(10, 5975)=158.49, CFI=.99, TLI=.99, RMSEA=.04, SRMR=.02 |
|  | **Sex** | **.23** | **.03** | **<.001** |  |  |
|  | Ethnicity | .03 | .01 | .05 |  |  |
|  | **BMI** | **.49** | **.02** | **<.001** |  |  |
|  | Income | .01 | .01 | .45 |  |  |
| *p-adjustment reported for all values relevant to hypothesis testing | | | | | | |

Supplementary Table 11: Cross-Sectional Regression Outcomes for Time-Use Sleep Characteristics and Restrictive Eating (N=2,150)

| Model | Predictor | Beta | SE | p | p (adjusted)* | Fit Indices |
| --- | --- | --- | --- | --- | --- | --- |
| Model 1 | Sleep Duration (Week) | -.01 | .02 | .61 | .89 | χ^2^(10, 2150)=33.68, CFI=.998, TLI=.996, RMSEA=.02, SRMR=.01 |
|  | **Sex** | **.23** | **.05** | **<.001** |  |  |
|  | Ethnicity | .02 | .03 | .41 |  |  |
|  | **BMI** | **.47** | **.04** | **<.001** |  |  |
|  | Income | -.03 | .02 | .14 |  |  |
| Model 2 | Sleep Duration (Weekend) | -.03 | .02 | .14 | .89 | χ^2^(10, 2150)=34.07, CFI=.998, TLI=.996, RMSEA=.02, SRMR=.01 |
|  | **Sex** | **.24** | **.05** | **<.001** |  |  |
|  | Ethnicity | .02 | .03 | .42 |  |  |
|  | **BMI** | **.47** | **.04** | **<.001** |  |  |
|  | Income | -.03 | .02 | .14 |  |  |
| Model 3 | Daytime Napping (Week) | .04 | .12 | .11 | .89 | χ^2^(10, 2150)=37.10, CFI=.998, TLI=.996, RMSEA=.03, SRMR=.02 |
|  | **Sex** | **.23** | **.05** | **<.001** |  |  |
|  | Ethnicity | .02 | .03 | .46 |  |  |
|  | **BMI** | **.47** | **.04** | **<.001** |  |  |
|  | Income | -.03 | .02 | .15 |  |  |
| Model 4 | Daytime Napping (Weekend) | -.004 | .12 | .86 | .89 | χ^2^(10, 2150)=33.89, CFI=.997, TLI=.99, RMSEA=.02, SRMR=.01 |
|  | **Sex** | **.24** | **.05** | **<.001** |  |  |
|  | Ethnicity | .02 | .03 | .41 |  |  |
|  | **BMI** | **.47** | **.04** | **<.001** |  |  |
|  | Income | -.03 | .02 | .15 |  |  |
| Model 5 | Night Awakening (Week) | .03 | .14 | .24 | .89 | χ^2^(10, 2150)=36.36, CFI=.998, TLI=.996, RMSEA=.02, SRMR=.02 |
|  | **Sex** | **.23** | **.05** | **<.001** |  |  |
|  | Ethnicity | .02 | .03 | .43 |  |  |
|  | **BMI** | **.47** | **.04** | **<.001** |  |  |
|  | Income | -.03 | .02 | .16 |  |  |
| Model 6 | Night Awakening (Weekend) | .02 | .13 | .37 | .89 | χ^2^(10, 2150)=35.34, CFI=.998, TLI=.996, RMSEA=.02, SRMR=.01 |
|  | **Sex** | **.23** | **.05** | **<.001** |  |  |
|  | Ethnicity | .02 | .03 | .41 |  |  |
|  | **BMI** | **.47** | **.04** | **<.001** |  |  |
|  | Income | -.03 | .02 | .17 |  |  |
| *p-adjustment reported for all values relevant to hypothesis testing | | | | | | |

Supplementary Table 12: Cross-Sectional Regression Outcomes for Overall Sleep Quality and Restrictive Eating at Age 17 (N=5975)

| Predictor | Beta | SE | p | Fit Indices |
| --- | --- | --- | --- | --- |
| Overall Sleep Quality | **.09** | **.02** | **<.001** | χ^2^(10, 5975)=254.43, CFI=.99, TLI=.98, RMSEA=.05, SRMR=.02 |
| Sex | **.32** | **.03** | **<.001** |  |
| Ethnicity | .003 | .01 | .84 |  |
| BMI | **.44** | **.02** | **<.001** |  |
| Income | **.06** | **.01** | **<.001** |  |

References:

Chen, F. F. (2007). Sensitivity of goodness of fit indexes to lack of measurement invariance. *Structural equation modeling: a multidisciplinary journal*, *14*(3),
 464-504.

Collings, P. J. (2021). Independent associations of sleep timing, duration and quality with adiposity and weight status in a national sample of adolescents:
 The UK Millennium Cohort Study. *Journal of Sleep Research*, *31*(1), e13436.

Evans, E. H., Adamson, A. J., Basterfield, L., Le Couteur, A., Reilly, J. K., Reilly, J. J., & Parkinson, K. N. (2017). Risk factors for eating disorder symptoms at 12
 years of age: A 6-year longitudinal cohort study. *Appetite*, *108*, 12-20.

Gerbing, D. W., Gerbing, M. D. W., & KernSmooth, S. (2023). Package ‘lessR’.

Isiordia, M., & Ferrer, E. (2018). Curve of factors model: A latent growth modeling approach for educational research. *Educational and Psychological
 Measurement*, *78*(2), 203-231.

Mackinnon, S., Curtis, R., & O'Connor, R. (2022). A tutorial in longitudinal measurement invariance and cross-lagged panel models using lavaan. *Meta-Psychology*, *6*.

Revelle, W., & Revelle, M. W. (2015). Package ‘psych’. *The comprehensive R archive network*, *337*(338).

Rosseel, Y. (2012). lavaan: An R package for structural equation modeling. *Journal of statistical software*, *48*, 1-36.

Van Buuren, S., & Groothuis-Oudshoorn, K. (2011). mice: Multivariate imputation by chained equations in R. *Journal of statistical software*, *45*, 1-67.

Wickham, H., Averick, M., Bryan, J., Chang, W., McGowan, L. D. A., François, R., ... & Yutani, H. (2019). Welcome to the Tidyverse. *Journal of open source
 software*, *4*(43), 1686.

1. Introduction of online assessment format within age 17 sweep (instead of in-person) [↑](#footnote-ref-1)
2. Less than 1% of missing values on individual items [↑](#footnote-ref-2)
3. For the final analysis, missing values were substituted for covariates based on information provided in previous sweeps [↑](#footnote-ref-3)
